# Influence of seasonal changes on disease activity and distribution of affected joints in rheumatoid arthritis

**DOI:** 10.1186/s12891-019-2418-2

**Published:** 2019-01-18

**Authors:** Hiroaki Mori, Tetsuji Sawada, Susumu Nishiyama, Kota Shimada, Koichiro Tahara, Haeru Hayashi, Eri Kato, Mayu Tago, Toshihiro Matsui, Shigeto Tohma

**Affiliations:** 1 Department of Rheumatology, Tokyo Medical University Hospital, 6-7-1 Nishi-Shinjuku, Shinjuku, Tokyo 160-0023 Japan; 2 Rheumatic Disease Center, Kurashiki Medical Center, 250 Bakuro, Kurashiki, Okayama 710-8522 Japan; 3 Department of Rheumatic Diseases, Tokyo Metropolitan Tama Medical Center, 2-28-29 Musashidai, Fuchu, Tokyo 183-8524 Japan; 4 Department of Rheumatology, Clinical Research Center for Allergy and Rheumatology, National Hospital Organization Sagamihara Hospital, 18-1 Sakuradai, Minami, Sagamihara, Kanagawa 252-0392 Japan; 5 Department of Rheumatology, National Hospital Organization Tokyo Hospital, 3-1-1 Takeoka, Kiyose, Tokyo, 204-8585 Japan

**Keywords:** Rheumatoid arthritis, Epidemiology, Seasonality, Disease activity

## Abstract

**Background:**

Previous studies suggest that RA activity is sensitive to seasonal changes. This study explored the influence of season on RA activity, particularly the distribution of affected joints, using a nationwide database in Japan.

**Methods:**

We investigated 12,839 patients whose RA activity was recorded in spring (*n* = 3250), summer (*n* = 916), fall (*n* = 1021), and winter (*n* = 7652). Disease activity score (DAS) 28-CRP, simplified disease activity index (SDAI), and clinical disease activity index (CDAI) were used as indices of disease activity. Disease activity was also assessed according to DAS28-CRP scores (remission, low, moderate, or high). The affected joint distribution was investigated using novel joint indices (***x***, ***y***, ***z***), where ***x*** and ***y*** are indices for the upper and lower joints, respectively, and ***z*** is the index for large joint predominance.

**Results:**

Mean DAS28-CRP and median SDAI and CDAI scores were highest in spring and lowest in fall. There was a significant difference in the DAS28-CRP for fall versus spring and winter. Fall was associated with a higher remission rate, and spring and winter with high and moderate RA activity, respectively. Significant differences in ***x***, ***y***, SDAI, and CDAI scores were found for spring versus summer, fall, and winter, in addition to fall versus winter (except in ***y***). There was no seasonal difference in the ***z*** index.

**Conclusions:**

RA activity in the upper and lower extremities may be highest in spring, followed by winter. Seasonal changes should be considered in patients with RA to better understand their symptoms.

## Background

Rheumatoid arthritis (RA) is a systemic autoimmune disease characterized by persistent synovitis that can lead to functional impairment. The activity of rheumatoid synovitis is known to fluctuate over time. Patberg et al. reviewed the literature on meteorological conditions [1] and found a significant relationship between weather and RA activity in 11 reports [2–12], indicating an important association of humidity with signs and symptoms of RA. However, no clear relationship was observed in 6 studies [13–18]. Subsequent studies have demonstrated that the impact of weather on RA activity is mediated by various factors, including temperature, humidity, sunshine, and atmospheric pressure [19–23], although some of these factors remain controversial [24, 25].

In many parts of the world, the year is divided into four 3-month seasons (winter, spring, summer, and fall) that are characterized by specific weather conditions (temperature, atmospheric pressure, humidity, and precipitation) and hours of daylight [26]. Previous studies have suggested that seasonal changes could influence the clinical manifestations of RA. For example, it has been reported that the onset of RA is more likely in winter than in summer in the northern hemisphere and that working in a cold environment increases the risk of developing RA [27]. Moreover, the onset of symptoms of arthritis during winter or spring was reported to be associated with more radiographic joint damage after 6 months in patients with early RA [28]; however, that report was followed by a conflicting one [29]. It is also widely believed that symptoms in patients with musculoskeletal pain are worse in winter [30]. Another study demonstrated that rheumatic symptoms were exacerbated by seasonal changes in approximately half of patients with rheumatic diseases, including RA [31]. In a study based on data from a large RA database in Japan, Iikuni et al. demonstrated that RA activity was higher in spring and lower in fall [32].

The hallmark of RA is symmetric polyarthritis affecting the small joints of the hands and feet [33], but the large joints (shoulders, elbows, knees, and ankles) and wrists may also be involved [34]. However, there have been no reports in the literature on the effects of changes in season on the distribution of affected joints in RA. Nishiyama’s joint indices (JIs; ***x***, ***y***, ***z***) are novel measures of RA that have 3 components: ***x*** and ***y*** are indices of RA disease activity in the upper and lower extremities, respectively, and ***z*** is an index of the predominance of large joint involvement [35, 36]. In this study, we assessed RA activity and the distribution of affected joints using the ***x***, ***y***, and ***z*** indices and data from a nationwide RA database in Japan (National Database of Rheumatic Diseases by iR-net in Japan, *NinJa*) [37]. Based on the data obtained, we discuss the influence of seasonal changes on the activity of RA.

## Methods

### Data source

*NinJa* is a nationwide, multicenter, observational database that was established in 2002 and contains data on RA patients treated in Japan [37]. RA patients at each participating medical institution are enrolled in *NinJa* at random. The clinical data of RA patients registered in *NinJa* are collected once a year from each patient at any time point during the indicated year. In this study, we used data from *NinJa*2016, which contains data for 15,341 patients with RA collected between April 1, 2016, and March 31, 2017. To analyze the effects of season on RA activity, we used the data from 12,839 patients for whom data on tender joint count (TJC), swollen joint count (SJC), 10-cm visual analog scale (VAS) pain score, patient’s global assessment of disease activity (PGA), physician’s global assessment of disease activity (PhGA), C-reactive protein (CRP) level, Steinbrocker classification of radiographic stage, and functional class were available. The cutoff age for defining the patients as older or younger was 65 years. The seasons were divided into spring (March, April, May), summer (June, July, August), fall (September, October, November), and winter (December, January, February), while acknowledging that weather conditions in Japan can be different even during the same season depending on the location of the participating institutions.

The *NinJa* study protocol was reviewed and approved by the ethics committee at each participating institution.

### Composite disease activity indices for RA

Disease activity score (DAS) 28-CRP, simplified disease activity index (SDAI), and clinical disease activity index (CDAI) were calculated from the TJC and SJC (28-joint count), PGA, and CRP [38]. RA activity was categorized according to DAS28-CRP score as remission (< 2.3), low (≤2.7), moderate (≤4.1), or high (> 4.1) [39].

### Novel joint index

We used the methodology previously described by Nishiyama et al. in order to measure RA activity and affected joint distribution using the joint indices consisting of 3 components (***x***, ***y***, ***z***) [35]. The indices ***x*** and ***y*** are the joint indices for upper and lower extremities, respectively. The index ***z*** reflects the predominance of large joints over small joints.

### Statistical analysis

The RA patients whose data were recorded in spring (*n* = 3250), summer (*n* = 916), fall (*n* = 1021), and winter (*n* = 7652) were all independent patients, so paired analyses were not required for the present study. For continuous variables with a normal distribution, one-way analysis of variance (ANOVA) was used to examine the differences in average values among the four seasons, followed by the post hoc Tukey-Kramer method for multiple comparisons. The Kruskal-Wallis test was used for continuous variables with a skewed distribution. Pearson’s chi-square test was used for categorical variables. A post hoc test based on adjusted standardized residuals (ASR) was used for multiple comparisons, whereby absolute values of ASR that were more than 1.96 and 2.58, respectively, were considered to correspond to significance levels of 0.05 and 0.01. The statistical analysis was performed using IBM SPSS Statistics version 24 software (IBM Corp., Armonk, NY) and JMP version 12.0.1 (SAS Institute Inc., Cary, NC). All significance levels were set at *p* < 0.05 (two-sided).

## Results

### Demographic and clinical data

In *NinJa*, clinical data are collected once per year from each participating patient at any time point during the indicated year. We used the clinical data for 2016 (*NinJa*2016), which were collected between April 1, 2016 and March 31, 2017. Data on TJC, SJC, VAS pain score, PtGA, PhGA, CRP, disease stage, and functional class were available for 12,839 patients with RA registered in *NinJa*2016. Of these 12,839 patients, 3250, 916, 1021, and 7652 were evaluated in spring, summer, fall, and winter, respectively. One-way ANOVA and Pearson’s chi-square tests did not reveal a statistically significant seasonal difference in age or sex distribution or in the proportion of patients with stage III–IV disease (Table 1). However, there were significant differences in disease duration and the proportion of patients with class 3–4 functional status. Post hoc analysis revealed that the disease duration was significantly longer in RA patients evaluated in fall than in those evaluated in winter (*p* < 0.05) and that the proportion of patients with class 3–4 functional status was significantly lower in patients evaluated in fall (*p* < 0.01) and higher in those evaluated in winter (*p* < 0.05).

**Table 1:** Demographic and clinical characteristics of patients with rheumatoid arthritis by season in *NinJa*2016

	Total	Spring	Summer	Fall	Winter	*p*-value^#^
	*n* = 12,839	*n* = 3250	*n* = 916	*n* = 1021	*n* = 7652	
Age (years)	65.2 ± 12.9	65.3 ± 13.1	65.5 ± 13.2	65.4 ± 12.8	65.1 ± 12.9	0.649
Female (%)	10,287 (80.1%)	2600 (80.0%)	746 (81.4%)	825 (80.8%)	6166 (79.9%)	0.680
Disease duration (years)	13.4 ± 11.0	13.4 ± 11.2	13.9 ± 11.4	14.2 ± 10.8	13.2 ± 10.9	0.026
Stage III–IV (%)	5917 (46.1%)	1456 (44.8%)	451 (49.2%)	481 (47.1%)	3529 (46.1%)	0.100
Class 3–4 (%)	2412 (18.8%)	613 (18.9%)	162 (17.7%)	153 (15.0%)	1484 (19.4%)	0.007
TJC	1.9 ± 4.2	2.0 ± 4.1	1.4 ± 3.4	1.6 ± 3.9	2.0 ± 4.3	< 0.001
SJC	1.4 ± 2.7	1.8 ± 3.3	1.4 ± 2.7	1.0 ± 2.3	1.3 ± 2.5	< 0.001
Pain (cm)	2.4 ± 2.3	2.4 ± 2.3	2.5 ± 2.3	2.3 ± 2.2	2.4 ± 2.2	0.463
PGA (cm)	2.5 ± 2.2	2.5 ± 2.2	2.6 ± 2.3	2.4 ± 2.2	2.5 ± 2.2	0.332
PhGA (cm)	1.5 ± 1.4	1.5 ± 1.5	1.4 ± 1.4	1.2 ± 1.3	1.5 ± 1.4	< 0.001
mHAQ	0.39 ± 0.61	0.41 ± 0.63	0.40 ± 0.62	0.38 ± 0.58	0.38 ± 0.61	0.126
CRP (mg/dL)	0.55 ± 1.21	0.56 ± 1.26	0.58 ± 1.14	0.55 ± 1.22	0.55 ± 1.20	0.854

^#^Chi-square tests were used for categorical variables and analysis of variance tests for continuous variables. *TJC* tender joint count, *SJC* swollen joint count, *PGA* patient global assessment of RA disease activity, *PhGA* physician global assessment of RA disease activity, *VAS* visual analog scale, *CRP* C-reactive protein, *mHAQ* modified Health Assessment Questionnaire

### Seasonal differences in ACR core set of disease activity variables

One-way ANOVA of the 7 parameters included in the ACR core set of measures used in clinical trials of RA (TJC, SJC, pain VAS score, PGA, PhGA, mHAQ, and CRP) revealed significant differences in the distributions of TJC, SJC, and PhGA (Table 1). A post hoc analysis using the Tukey-Kramer method revealed significant differences in the distribution of TJC in summer versus spring (*p* = 0.001) and winter (*p* < 0.001) as well as in fall versus winter (*p* = 0.013); the difference between fall and spring did not reach statistical significance (*p* = 0.054). There were statistically significant differences in the SJC for fall versus spring (*p* < 0.001), summer (*p* = 0.019), and winter (*p* = 0.015), in addition to spring versus summer (*p* < 0.001) and winter (p < 0.001). There were also statistically significant differences in PhGA for fall versus spring (*p* < 0.001) and winter (*p* < 0.001) in addition to spring versus summer (*p* = 0.003).

### Seasonal differences in composite RA activity indices

DAS28-CRP, SDAI, and CDAI scores were highest in spring and lowest in fall. A post hoc analysis using the Tukey-Kramer method revealed statistically significant differences in the distribution of DAS28-CRP scores in fall versus spring (*p* < 0.001) and winter (*p* = 0.004; Table 2). There were also significant differences in the SDAI scores for spring versus summer (*p* = 0.021), fall (*p* < 0.001), and winter (*p* = 0.011) in addition to fall versus winter (*p* = 0.001). Similarly, there were significant differences in the distribution of CDAI scores for spring versus summer (*p* = 0.009), fall (*p* < 0.001), and winter (*p* = 0.007) as well as fall versus winter (*p* < 0.001).

**Table 2:** Values of composite indices of disease activity in patients with rheumatoid arthritis by season in *NinJa*2016

	Total	Spring	Summer	Fall	Winter	*p*-value^#^
	*n* = 12,839	*n* = 3250	*n* = 916	*n* = 1021	*n* = 7652	
DAS28-CRP	2.3 ± 1.0	2.4 ± 1.1	2.3 ± 1.0	2.2 ± 1.0	2.3 ± 1.0	0.001
SDAI	5.1 [2.1, 9.7]	5.4 [2.1,10.2]	4.8 [1.9, 9.4]	4.3 [1.7, 8.1]	5.1 [2.1, 9.8]	< 0.001
CDAI	4.7 [1.8, 9.0]	5.0 [1.9, 9.7]	4.3 [1.6, 8.6]	4.0 [1.5, 7.6]	4.7 [1.9, 9]	< 0.001

^#^Analysis of variance was used for the DAS28-CRP score. The Kruskal-Wallis test was used for the SDAI and CDAI scores. *DAS28* Disease Activity Score with 28-joint counts (average value ± standard deviation), *SDAI* simplified disease activity index (median [interquartile range]), *CDAI* clinical disease activity index (median [interquartile range])

**Fig. 1:**
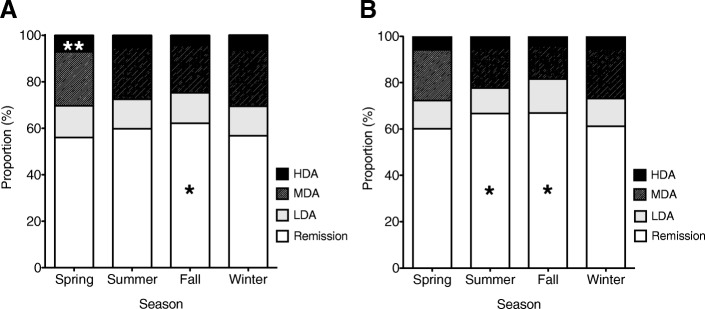
Seasonal breakdown of rheumatoid arthritis (RA) disease activity according to DAS28-CRP score in 12,839 patients with RA (**a**) and in a subgroup of 5255 younger patients (< 65 years) (**b**) in *Ninja*2016. The proportions of patients categorized as having remission (DAS28-CRP < 2.3), low disease activity (LDA, 2.3 ≤ DAS28-CRP < 2.7), moderate disease activity (MDA, 2.7 < DAS28-CRP ≤4.1), and high disease activity (HAD, DAS28-CRP ≥4.1) in each season are shown in a stacked bar graph. *p < 0.01, **p < 0.05

We also analyzed the influence of seasonal changes on RA activity by categorizing the disease activity as remission, low, moderate, or high based on DAS28-CRP scores. There was a significantly higher proportion of patients in remission (*p* < 0.01) and a significantly lower proportion of patients with moderate disease activity (*p* < 0.01) in fall (Fig. 1 a). In contrast, spring was associated with high disease activity (*p* < 0.01) and winter with moderate disease activity (*p* < 0.01). We divided the RA patients into an older group (≥65 years, *n* = 7584) and a younger group (< 65 years; *n* = 5255) to examine the influence of age on seasonal variations in RA disease activity. We found a significantly higher proportion of patients in remission in fall (*p* < 0.01) and summer (*p* < 0.01) and a significantly lower proportion of patients with moderate disease activity in fall (*p* < 0.01; Fig. 1b).

### Seasonal differences in joint distribution

The ***x*** and ***y*** indices reflect the RA activity in the upper and lower extremities, respectively. The ***z*** value is an indicator of predominant involvement of large joints over small joints (JI of large joints minus that of small joints).

**Fig. 2:**
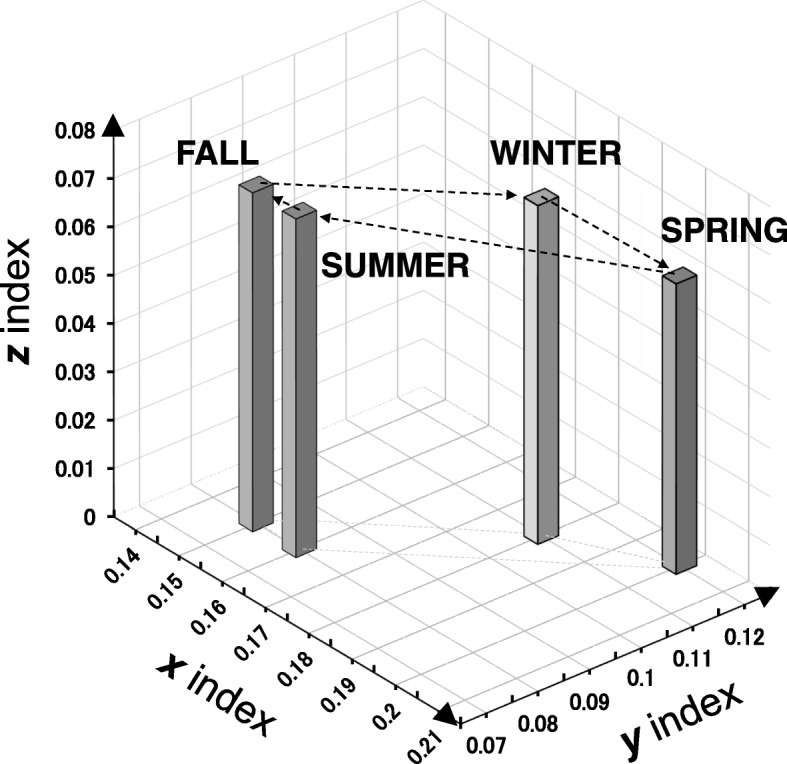
Average values for joint index ***x*** (upper joint), ***y*** (lower joint), and ***z*** (large joint index) plotted by season in *NinJa*2016. Average ***x***, ***y***, and ***z*** values for the four seasons are plotted in a three-dimensional format, where ***x*** and ***y*** are plotted horizontally and ***z*** is plotted vertically. The average ± standard deviation values for ***x***, ***y***, and ***z*** in each season are as follows: spring, 0.20 ± 0.31, 0.12 ± 0.26, and 0.39 ± 0.43; summer, 0.16 ± 0.28, 0.08 ± 0.23, and 0.34 ± 0.44; fall, 0.15 ± 0.26, 0.08 ± 0.23, and 0.35 ± 0.44; and winter, 0.18 ± 0.29, 0.11 ± 0.26, and 0.38 ± 0.44. Each bar is connected by dotted arrows in the order of the four seasons for clarity

The average ***x***, ***y*** and ***z*** values are plotted in Fig. 2. There were significant differences in these values for spring versus summer (*p* = 0.001), fall (*p* < 0.001), and winter (*p* = 0.003) as well as for fall versus winter (*p* = 0.006). There were significant differences in ***y*** for spring versus summer (*p* = 0.004) and fall (*p* = 0.002) as well as for fall versus winter (*p* = 0.037); the difference between spring and winter did not reach statistical significance (*p* = 0.25). There was no significant seasonal difference in the ***z*** value (0.06 ± 0.30, 0.07 ± 0.28, 0.07 ± 0.25, and 0.07 ± 0.28 in the order of spring to winter).

## Discussion

In this study, using a nationwide RA database (*NinJa*2016) in Japan, we investigated the influence of season on clinical parameters of RA, including TJC, SJC, VAS pain score, PGA, PhGA, CRP, stage, and functional class data, for 12,839 patients with RA. Our results show a significant difference in RA activity among the seasons. Furthermore, we have demonstrated for the first time that arthritic symptoms are affected by seasonal change in both the upper and lower extremities, as assessed by a newly developed joint index.

Iikuni et al. previously demonstrated that RA activity, evaluated both subjectively and objectively, is higher in spring and lower in fall (*n* = 1665) using the large IORRA (Institute of Rheumatology, Rheumatoid Arthritis) cohort database, from which they retrieved clinical information for registered patients with RA in spring and fall for 5 consecutive years [32]. Sadamoto et al. also investigated the relationship between RA activity and seasonal change in 280 patients with RA in Japan, and found that 63% of patients recognized seasonal deterioration, which was frequent in spring and winter [40]. Consistent with their findings, we found that fall was associated with a high proportion of patients in remission as well as a low proportion of patients with moderate disease activity, whereas spring and winter were significantly associated with moderate and high disease activity, respectively. Furthermore, DAS28-CRP scores were numerically highest in spring, followed by winter, summer, and fall. Although a significant seasonal difference in DAS28-CRP scores was observed only in comparisons between fall and spring and between fall and winter, the SDAI and CDAI scores were significantly higher in spring than in summer, fall, or winter. The SDAI and CDAI scores were also significantly higher in winter than in fall. Therefore, it was considered that spring was the season associated with the highest RA activity.

In this study, we analyzed the influence of seasonal changes on the distribution of affected joints in patients with RA using the newly developed Nishiyama’s joint index [35]. To our knowledge, this is the first study to do so. We demonstrated that the ***x*** index was numerically elevated in the order of spring, winter, summer, and fall. A statistically significant difference in the ***x*** index was found between spring and the other seasons and between winter and fall. A similar statistically significant seasonal pattern was observed for the ***y*** index, except between spring and winter. Given that the ***x*** and ***y*** indices reflect RA activity in the upper and lower extremities, respectively, it was considered that the RA activity in both the upper and lower extremities was greater in spring, presumably followed by winter. However, there was no significant change in the ***z*** index, suggesting that large and small joints were similarly affected, irrespective of season.

It is unknown why RA activity was higher in spring and winter in our study, as in earlier reports. Winter is generally associated with an increase in musculoskeletal symptoms [41], partly because cold weather is likely to cause an increase in muscle spasm and a decrease in the blood circulation. In a longitudinal observational study of 133 patients with RA, Savage et al. demonstrated a significant decrease in disease activity in conditions of more sunshine and low humidity [21]. With regard to spring, it should be noted that individuals with chronic pain may be vulnerable to changes in the local climate [42]. Japan has a unique climate pattern in early spring known as *san-kan-shi-on* (3 days of cold weather followed by 4 days of warm weather) because of alternating high and low atmospheric air pressure systems over a period of approximately 7 days [43]. Of interest, Terao et al. have recently investigated the relationship between atmospheric air pressure and RA activity using data for 2131 patients with RA in the KURAMA (Kyoto University Rheumatoid Arthritis Management Alliance) database and demonstrated an inverse association of air pressure with activity of rheumatoid synovitis. This finding raises the possibility that rapid and repeated changes in local atmospheric pressure could lead to unstable RA and subsequent deterioration of disease activity during the *san-kan-shi-on* period in spring. Alternatively, it is possible that this high disease activity is triggered by stressful life events that coincide with spring. The financial year starts in April in Japan, so spring is the season in which patients with RA may feel the most financial stress. Other events likely to occur in spring include changes in lifestyle, sitting entrance examinations, which are anxiety-provoking for both parents and children, and changes in personnel in the workplace. RA activity is known to be affected by psychological distress [44] and could be elevated in spring because of these stressful events.

Previous studies have demonstrated that elderly individuals are generally more likely to experience frequent and prolonged pain than their younger counterparts [45, 46]. However, in the present study, there was a significant seasonal variation in RA disease activity in younger RA patients as well, and significant associations of fall with a higher proportion of remission and a lower proportion of moderate disease activity. Therefore, we need to recognize that seasonal changes in RA activity may occur in any age group.

The biological mechanism for seasonal variation in RA activity remains to be elucidated. Vitamin D metabolism might be a contributing factor [47–49], given that vitamin D has effects on innate and acquired immunity [47] and exposure to sunlight is required for ultraviolet B-induced synthesis of vitamin D in the skin. Ishikawa et al. demonstrated that serum vitamin D levels follow a lagged pattern relative to the astronomical seasons, peaking in late summer (August) and troughing in late winter (February) [47], possibly as a result of fewer daylight hours and less outdoor activity because of cool or cold weather. A meta-analysis by Lee et al. demonstrated that vitamin D deficiency is prevalent in patients with RA and found a significant inverse correlation between low serum vitamin D levels and RA activity in these patients [50], suggesting that hypovitaminosis D could play an important role in the seasonality of RA activity. Furthermore, in a comprehensive whole-transcriptome analysis of peripheral blood mononuclear cells in vitro, Dopico et al. demonstrated significant and widespread seasonal changes in the immune system, including a predominance of pro-inflammatory gene expression in peripheral blood mononuclear cells in winter as compared with summer [51]. Their findings suggest that seasonality of RA activity might be explained at least in part by seasonal changes in transcriptional signatures within the immune system.

This study has several limitations that need to be addressed. First, the patients with RA in *NinJa* were evaluated once a year at any time point at the discretion of their physicians, which enabled us to study the seasonal influence on RA activity. However, this was not a longitudinal study that examined changes in disease activity in a cohort of RA patients over a defined period of time. Therefore, one limitation of this study is its cross-sectional unpaired design, which could potentially have introduced bias. Second, although the age at RA onset has increased significantly over the last 10 years in Japan [52], *NinJa* does not collect data on concomitant diseases, such as osteoarthritis. Timmermans et al. demonstrated that elderly patients with osteoarthritis often perceived the weather as affecting their pain [53]. In the present study, younger patients were also vulnerable to seasonal changes in RA activity, suggesting that symptoms of osteoarthritis do not play a major role in seasonal variation in RA activity. Nevertheless, we cannot exclude the possibility that unidentified comorbidities, such as osteoarthritis, might be confounding factors contributing to seasonal variation in RA activity. Third, the study did not include a control group for comparison, such as patients with osteoarthritis or low back pain. Therefore, it is unclear whether or not the observed seasonal influence is specific for RA or includes musculoskeletal diseases other than RA. Fourth, there is a regional difference in terms of the periods of the four seasons in Japan, which is located at a latitude of 24°–46°N and a longitude of 123°–146°E. If we had defined the period of each individual season corresponding to each region based on local weather conditions, a more significant seasonal influence on RA activity might have been found. It should also be noted that there were significant differences in disease duration and the proportion of patients with class 3–4 functional status according to season, which could have affected our observations. With respect to functional impairment, we consider it possible that the modest RA activity in fall improved the functional status of our study population, resulting in a decreased proportion in functional class 3–4. The difference in disease duration among the seasons (ranging from 13.2 years in winter to 14.2 years in fall) appears to be clinically insignificant, given that functional impairment was reported to develop most rapidly during the first year after disease onset, with a slow and almost linear rate of increase thereafter [54]. However, this study has some strengths, including the relatively large patient population studied and use of a novel joint index that enabled us to demonstrate a significant influence of season on the distribution of affected joints in patients with RA.

## Conclusions

We have demonstrated an effect of seasonality on disease activity and affected joint distribution in patients with RA using a nationwide RA database in Japan. Health care providers should be aware of seasonal fluctuations in the manifestations of RA, particularly the functional deterioration that can occur in winter and spring, and help patients with the disease to adjust to seasonal changes in terms of their activities of daily living (e.g., by suggesting they wear warmer clothes in the cold weather and temporarily increasing their doses of analgesic medication if necessary). A large-scale, longitudinal study of the relationship between daily weather conditions and RA activity, including patient-reported outcomes, is needed to clarify how seasonal changes and other meteorological factors affect rheumatoid synovitis and to explore new anti-rheumatic interventions.

## Abbreviations

ASR: Adjusted standardized residuals
CDAI: Clinical disease activity index
CRP: C-reactive protein
DAS28-CRP: Disease activity score 28-CRP
JI: Joint index
mHAQ: Modified health assessment questionnaire
*NinJa*: National Database of Rheumatic Diseases by iR-net in Japan
PBMC: Peripheral blood mononuclear cells
PGA: Patient’s global assessment of disease activity
PhGA: Physician’s global assessment of disease activity
RA: Rheumatoid arthritis
SDAI: Simplified disease activity index
SJC: Swollen joint count
TJC: Tender joint count
VAS: Visual analog scale
